# Disparities in Basic Housing Needs as a Predictor of Psychological Well-Being Among Older Adults

**DOI:** 10.1093/geroni/igab046.2354

**Published:** 2021-12-17

**Authors:** Meghan Custis, Jeongeun Lee, Natasha Peterson

**Affiliations:** 1 Iowa State University, MUSCATINE, Iowa, United States; 2 Iowa State University, Ames, Iowa, United States; 3 Iowa State University, Iowa State University, Iowa, United States

## Abstract

Adequate housing and safe environments are among older adults’ foundational needs. Prior research suggests minority older adults face significant barriers to accessing affordable and appropriate housing. However, the effects of this environmental press on their psychological well-being are rarely addressed. This project examined racial disparities between minority and white older adults’ housing and environment conditions and the differential impact on their psychological well-being. Using nationally representative data from the National Health & Aging Trends Study (NHATS), older adults' reported rating of the quality of housing conditions, financial security, neighborhood security, and the interviewer’s rating of the home environment were analyzed. A total of 4,048 community-dwelling older adults aged 65 and over were selected for analysis. The sample demographics are predominantly white (77.5%), female (61.4%), and residing in the community (82%). Results found minority older adults reported poorer housing conditions, fewer home modifications, and lower financial and neighborhood security, compared to white counterparts. The impact of housing quality was more detrimental to minority older adults’ psychological well-being, compared to white counterparts. These findings suggest a significant negative impact of home conditions on the psychological well-being of minority older adults. Home modifications are a viable option to increase or preserve functional status in the home, which could lessen the deleterious effects of environmental press on older adults’ psychological outcomes, especially minorities. This study’s findings provide information that bolsters our knowledge of housing and environment conditions, which are critical in efforts to reduce health disparities in late life.

